# Potential protection by vitamin D against DNA fragmentation and bone marrow cytotoxicity induced by chloramphenicol

**DOI:** 10.1016/j.toxrep.2024.101828

**Published:** 2024-11-22

**Authors:** Nagla Zaky Ibrahim El-Alfy, Asmaa Ahmed Khaled Emam, Mahmoud Fathy Mahmoud, Omnia Nabeel Mohamed Morgan, Sally Ramadan Gabr Eid El-Ashry

**Affiliations:** Biological and Geological Sciences Department, Faculty of Education, Ain Shams University, El-Khalyfa El-Mamoun Street Abbasya, Cairo 11341, Egypt

**Keywords:** Chloramphenicol, Vitamin D, DNA Fragmentation, Micronucleus, Liver cells, Bone marrow cells

## Abstract

Vitamin D (Vit D) has gained significant attention in health research recently as a result of its potential protective effects against various cellular damages. This study aimed to investigate the ability of vitamin D to mitigate deoxyribonucleic acid (DNA) fragmentation in liver cells and bone marrow cytotoxicity induced by chloramphenicol (CAP). Sixty male albino mice were divided into six groups: control, chloramphenicol-treated (250 and 500 mg/kg body weight, 5 days per week for 4 weeks), vitamin D-treated (800 IU/kg body weight, 5 days per week for 4 weeks) and vitamin D plus chloramphenicol-treated groups. Results of DNA fragmentation test revealed that oral treatment with low and high doses of CAP significantly increased the frequency of DNA fragmentation in liver cells in comparison with the control, whereas oral treatment with vitamin D alone or plus low and high doses of chloramphenicol significantly reduced the genotoxicity in liver cells in comparison with the control group. Micronucleus analysis showed that CAP treatment at low and high doses significantly increased micronuclei formation and cytotoxicity in bone marrow cells. However, vitamin D significantly reduced the micronuclei formation in bone marrow cells of mice treated with chloramphenicol. Vitamin D alone showed no significant difference in the frequency of micronuclei and bone marrow cytotoxicity compared to the control group. Accordingly, further research exploring the mechanisms underlying the protective effects of vitamin D and investigating optimal dosing regimens is warranted. Also, clinical studies evaluating the efficacy of vitamin D supplementation to mitigate the adverse effects of chloramphenicol in human patients are recommended.

## Highlights

• Vitamin D reduces chloramphenicol (CAP)-induced deoxyribonucleic acid (DNA) fragmentation in liver cells.
• CAP increases micronuclei in bone marrow; Vitamin D mitigates this.
• Vitamin D shows cytoprotective effects against CAP toxicity.
• Study supports Vitamin D supplementation with CAP treatment.
• Further research on Vitamin D's protective mechanisms is needed.

## Graphical Abstract

**Figure:**
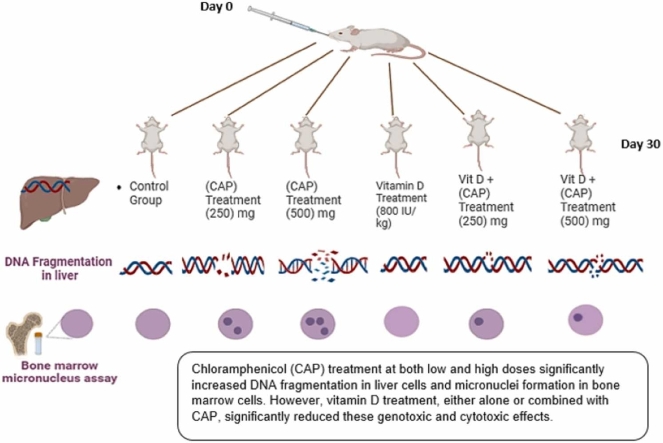

## 1 Introduction

In recent years, the escalation of multidrug-resistant (MDR) bacteria has surpassed the progress in creating novel, potent antibiotics. Consequently, there has been a reassessment of antimicrobial agents that were previously discarded [1]. Among these agents, chloramphenicol (CAP) has garnered attention for its broad-spectrum activity against both Gram-positive and Gram-negative microorganisms, making it a valuable tool in the management of severe infections such as typhoid fever and meningitis [2]. The chloramphenicol class of antibiotics encompasses chloramphenicol (CAP) along with its analogs, thiamphenicol (TAP), and florfenicol (FF) [3].

Chloramphenicol (CAP) is an antibiotic with broad-spectrum bacteriostatic properties, originally isolated from *Streptomyces venezuelae* in 1947 during the heyday of antibiotic exploration, often referred to as the "golden era" of antibiotic discovery [4]. It enjoyed widespread use globally in both human and veterinary medicine. However, contemporary regulations have restricted the utilization of CAP in food production, particularly within the animal food-production chain. Chloramphenicol inhibits protein synthesis in bacteria by binding to the bacterial ribosomal subunit. This interference with protein formation leads to bacterial death [5]. Due to its advantages of relatively low production cost and high stability, chloramphenicol has gained widespread acceptance for use in developing countries [6]. However, its use as a human medication has significantly declined in many regions for reasons elaborated below. In contrast, certain Asian countries continue to employ CAP for human therapeutic purposes. Notably, ophthalmic infections still receive extensive treatment with CAP [7].

Bale et al. [8] reported that CAP is formulated as eye drops for the treatment of ophthalmic infections. Ophthalmic chloramphenicol is valuable as an over-the-counter medication, providing rapid relief from the discomfort of ocular infections and minimizing the necessity for clinical consultations for mild ophthalmic infections**.** Despite its efficacy, numerous resistance mechanisms to CAP have been identified. However, the primary constraint on the clinical utility of CAP stems from its adverse effects, notably neurotoxicity, and hematologic disorders [9]. There is reasonable anticipation that chloramphenicol may act as a human carcinogen, supported by limited evidence of carcinogenicity observed in human studies. Previous study reported that the administration of chloramphenicol to mice led to an elevated occurrence of lymphoma in two strains and liver tumors in one strain [10].

From a biological perspective, CAP can induce two types of toxic manifestations in the bone marrow. The more frequently observed manifestation is bone marrow depression, characterized by reversibility, dose dependence and concurrent occurrence during antibiotic administration. The other manifestation is bone marrow aplasia, which is irreversible, often fatal, and may occur at times, typically weeks or months after discontinuation of antibiotic administration [11], The predominant toxic effects observed following the administration of CAP in eukaryotes can be elucidated by the demonstrated inhibitions of mitochondrial function and protein synthesis. Sall et al. [12] reported that exposure to chloramphenicol may potentially lead to significant impacts on both the environment and human health, intensified by recent research. Wiest et al. [13] demonstrated that chloramphenicol should be reserved for situations involving life-threatening infections where no other safer antibiotic is viable. The initial dosage should be determined by considering the patient's age and body weight. In cases where patients are severely ill and have accompanying liver dysfunction, it is advisable to reduce the dosage.

Chloramphenicol hinders messenger ribonucleic acid (mRNA) translation through the 70S ribosomes of prokaryotes, with no impact on 80S eukaryotic ribosomes. The majority of mitochondrial proteins are encoded by nuclear DNA and are transported from the cytosol, where they are synthesized, into the organelles. Although mitochondria can independently translate a limited number of proteins encoded by the mitochondrial genome using their ribosomes, these ribosomes share similarities with those of bacteria due to their prokaryotic origin. Consequently, chloramphenicol disrupts protein synthesis by mitochondrial ribosomes, and it is believed that chloramphenicol-induced anemia arises from this inhibition [14]. This condition is associated with deficiencies in the production of both red and white blood cells, resulting in a blood disorder and a heightened mortality rate [15].

Being a protein-synthesis inhibitor, CAP has the potential to disrupt the synthesis of immunoglobulin, with multiplying cells being the most adversely impacted. This phenomenon could explain the exhaustion and aplasia observed in both humans and animals after the administration of CAP [16]. Certainly, this antibiotic has been detected with high frequency in rivers, lakes, and seawaters, primarily due to the runoff from livestock farming and aquaculture activities [17].

Vitamin D, a steroid, has been consistently associated with the regulation of calcium homeostasis and bone mineralization [18]. Vitamin D can be obtained from three main sources: nutrition, ultraviolet B (UVB)-triggered internal production and supplementation. In humans, the predominant synthesis of vitamin D occurs in the skin upon UVB exposure., while a minimal proportion is obtained from dietary sources [19]. When the human skin is exposed to UVB, cholecalciferol is synthesized from 7-dihydrocholesterol. Cholecalciferol, initially biologically inert, promptly attaches to vitamin binding proteins or albumin [20]. Subsequently, it enters the bloodstream and undergoes hydroxylation in the liver through the enzymatic actions of Cytochrome P450 Family 2 Subfamily R Member 1 (CYP2R1) and Cytochrome P450 Family 27 Subfamily A Member 1 (CYP27A1) which are protein coding genes. This process yields the inactive form known as 25-hydroxyvitamin D (25(OH)D), which serves as the primary circulating vitamin D metabolite and the most reliable indicator for determining vitamin D status in humans [21].

Systematic reviews suggest that vitamin D supplementation benefits the prevention of respiratory infections and enhances pulmonary function in vitamin D-deficient patients. In the context of viral respiratory infections, including coronavirus disease 2019 (COVID-19), maintaining optimal vitamin D levels may play a crucial role in immunoregulatory functions and mitigating exaggerated cytokine responses [22]. Beyond its traditional functions in maintaining good health, vitamin D has demonstrated a variety of anti-cancer and immune-modulating properties. Additionally, vitamin D serves various functions beyond skeletal health.

The recognition of widespread expression of the vitamin D Receptor (VDR) not only in the kidneys but also in the breast, prostate, brain, colon, macrophages, and other tissues highlights the diverse and intricate biological roles of vitamin D within the human body [23]. Vitamin D plays a secondary role in preventing DNA damage by regulating poly-ADP-ribose polymerase activity in the DNA damage response pathway, which is crucial for detecting DNA lesions. It also regulates the cell cycle to hinder the propagation of damaged DNA and control apoptosis to promote cell death. Although there is limited evidence to suggest that the prevention of DNA damage mediates the potential contribution of vitamin D to preventing human colorectal cancer, its exact mechanism remains unclear [24].

Calcitriol has been observed to inhibit proliferation, induce apoptosis, decrease angiogenesis, and enhance cell sensitivity to chemotherapy in various cancers and autoimmune diseases [25]. Considering the significant adverse effects associated with chemotherapy and other existing anti-cancer treatments, there is a growing interest in exploring the clinical application of vitamin D. This interest stems from the fact that its primary toxicity, hypercalcemia, can be mitigated by using vitamin D analogs [25]. Supplementation with a daily dose of 2000 IU of vitamin D has been shown to reduce the level of DNA damage, with this effect being particularly notable in patients with type 2 diabetes mellitus [26]. The potential of vitamin D in mitigating adverse effects and enhancing therapeutic outcomes warrants further investigation and clinical exploration.

## 2 Materials and methods

### 2.1 Animals

Male CD-1 albino mice (*Mus musculus*), aged between 6 and 8 weeks and weighing around 25±5 g, were sourced from Theodor Bilharz Research Institute in Cairo. After acquisition, they were housed in standard polyethylene cages and allowed to acclimate to the environment for a week before the commencement of the study. During this period, they had unrestricted access to water and a standard pellet diet. The ambient conditions were maintained at 25 ± 2 °C, with a relative humidity of 55 ± 10 %, and a 12-hour light/dark cycle. The study and all experimental procedures received approval from the Experimental Animal Care and Research Ethics Committee of Aims Shams University (Approval No. sci1332312004).

### 2.2 Chemicals

The chemicals employed in this study were chloramphenicol (CAP) and vitamin D. CAP procured from Egyptian pharmacies under the commercial name "Cidocetine," produced by Chemical Industries Development “CID” – A.R.E. Cidocetine, classified as an antibiotic, was obtained in the form of 250 mg chloramphenicol capsules. It is stored at temperatures not exceeding 30 degrees Celsius with a relative humidity below 70 %. To achieve the required concentrations (250 and 500 mg/kg b. wt.), a freshly prepared solution was made immediately before treatment by diluting it with distilled water. The solution was carefully shielded from light and heat during preparation. Vitamin D used in the study was sourced from Egyptian pharmacies under the commercial name " VIDROP," manufactured by Medical Union Pharmaceuticals (MUP) – Egypt. VIDROP is an oral solution containing cholecalciferol at a concentration of 2800 I.U. per milliliter, available in a 15 ml bottle. It is recommended to store VIDROP at temperatures not exceeding 30 degrees Celsius and to protect it from light.

### 2.3 Experimental design

Sixty mice were randomly divided into six groups, with each group containing ten animals. The groups were delineated as follows: **Group 1** served as the control and received 0.25 ml of sterile distilled water orally as a solvent. **Group 2** received an oral treatment of vitamin D at a dose of 800 IU/kg body weight (b. wt.) for five consecutive days per week over a span of four weeks. **Group 3** was treated orally with a low dose of CAP (250 mg/kg b. wt.) dissolved in 0.25 ml of sterile distilled water, administered for five consecutive days per week over a span of four weeks**. Group 4** received a protective dose of vitamin D one hour prior to the administration of the low dose of CAP, given for five consecutive days per week over a span of four week. **Group 5** was given a high dose of CAP (500 mg/kg b. wt.) dissolved in 10 ml of sterile distilled water, with the regimen involving oral administration for five consecutive days per week over a period of four weeks. **Group 6** received vitamin D prior to the administration of the high dose of CAP dissolved in 0.25 ml of sterile distilled water, for five consecutive days per week over four weeks. Vitamin D was administered to rats one hour prior to CAP treatment with same dose and duration mentioned. Both the control and treated animals were euthanized by cervical dislocation 24 hours after the last treatment, and samples were collected for further analysis.

### 2.4 DNA fragmentation test using agarose gel electrophoresis

This method is employed for the separation of distinct DNA fragments based on their respective sizes and charges. DNA samples are positioned in wells resembling pockets, representing the negative electrode. Subsequently, the fragments migrate towards the positive electrode within the electrical field. This procedure takes place within an agarose gel medium derived from seaweed. Larger fragments are situated closer to the wells, while the smallest fragments move towards the positive electrode. The sizes of DNA bands (fragments) are then compared to the known sizes of a DNA ladder. DNA extraction from tissue homogenates (200 mg) was performed using **the Zymo Research Quick-g DNA™ Mini Prep kit, Catalog No. D3024**. Following centrifugation of tissue homogenates at 12,000 g for 10 minutes at 4 °C, residual supernatants were utilized for DNA isolation.

### 2.5 Micronucleus test

In this study, Schmid's standard procedure was used with a minor modification [27]. Instead of employing fetal calf serum, a 5 % solution of bovine albumin sourced from the National Research Center in Giza, Egypt, was utilized as the suspending medium for bone marrow collection [28], [29]**.**

A screening of two thousand polychromatic erythrocytes (PCEs) per animal was conducted, and micronucleated PCEs (MNPCEs) were recorded. Additionally, identified normochromatic erythrocytes (NCEs) were also recorded. Finally, the percentage of MNPCEs and the PCE/NCE ratio were calculated for each animal.

### 2.6 Data analysis

The statistical evaluations were conducted utilizing the SPSS/computer PC program (version 16.0). Results were presented in terms of mean and standard deviation (mean ± SD). To assess the significance of differences between the two groups, the independent samples T-test was employed, determining whether the observed distinctions were statistically significant or attributable to sampling error. A significance level of (P< 0.05) was regarded as significant, while a significance level of P < 0.01 was considered a stronger level of significance. Conversely, a (P> 0.05) value was considered insignificant. The graphical representation was created using Excel 2019.

## 3 Results

### 3.1 DNA fragmentation test

The study revealed clear variations in the sizes of DNA fragments, signaling the degree of genotoxicity resulting from exposure to chloramphenicol. To conclude size, a reference DNA ladder with predetermined fragment sizes was utilized, facilitating the movement of DNA fragments through an agarose gel, aided by the application of an electrical field, making the process of formation of visible bands possible. The increased compactness of the bands implies a higher abundance of DNA fragments. Chloramphenicol administration to mice led to DNA fragmentation in hepatocytes at all tested doses, both with and without vitamin D. The genotoxic impact was particularly heightened at 500 mg/kg in the absence of vitamin D. The cohort subjected to a substantial dosage of 500 mg/kg without vitamin exhibited a notably significant (p<0.01) rise in DNA fragmentation, primarily in the lower size range of 100–3000 base pairs. Conversely, in the group pre-treated with vitamin D and then exposed to the same high dosage of CAP, there was a significantly marked (p<0.01) increase in the consolidation (band density) of DNA fragments, predominantly within the size range of 800–3000 base pairs.

**Fig. 1:**
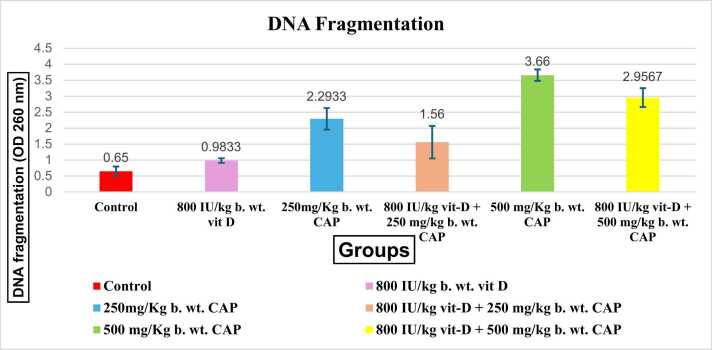
Chart representing the (Mean ± SD) of DNA fragmentation showing the comparison between the control and each treated group where samples treated with Vit D showed a decreased level of DNA fragmentation compared to the groups orally injected with CAP only.

**Fig. 2:**
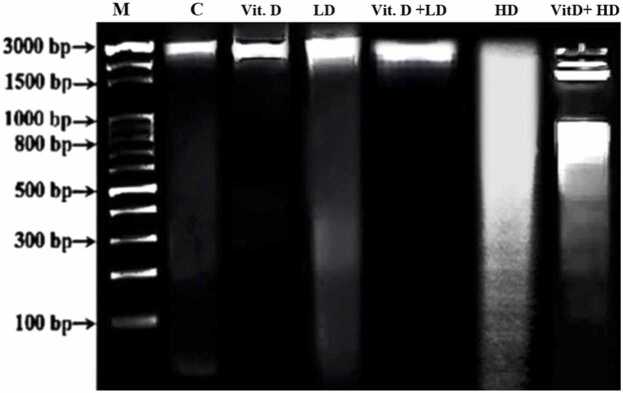
Photomicrograph showing the size of DNA fragments from liver tissue samples of male albino mice (*Mus musculus*), examined by agarose gel electrophoresis. Lane 1 (M) represents the DNA molecular weight marker, lane 2 (C) represents the control group, lane 3 (vit D) represents the group treated with vitamin D alone, lane 4 (LD) represents the group treated with a low dose of CAP without vitamin D, lane 5 (Vit D + LD) represents the group treated with vitamin D prior to a low dose of CAP, lane 6 (HD) represents the group treated with a high dose of CAP without vitamin D, and lane 7 (Vit D + HD) represents the group treated with vitamin D one hour before a high dose of CAP.

Similarly, administering low dose of CAP solely showed a significant increase (P<0.01) in DNA fragmentation bp with a monitored high density of bands, meanwhile, vit D + the low dose demonstrated a significant increase (p<0.005) in DNA compared to control group (Figs 1 and 2).

In the group treated with 800 IU/kg of vitamin D prior to a low dose of chloramphenicol (CAP), the DNA fragments ranged in size from approximately 3000 to 2000 base pairs (bp). Conversely, the group treated with a low dose of CAP without vitamin D exhibited DNA fragments ranging from ≤3000–1500 bp, indicating higher DNA damage levels, as evidenced by the higher density and larger fragment sizes.

Similarly, in the group treated with 800 IU/kg of vitamin D followed by a high dose of CAP, the DNA fragments ranged from approximately 3000 to 800 bp. In contrast, the group treated with a high dose of CAP without vitamin D showed DNA fragments ranging from ≤3000–100 bp. This also indicated increased DNA damage, with higher density bands suggesting the presence of larger DNA fragments.

### 3.2 Micronucleus test

**Fig. 3:**
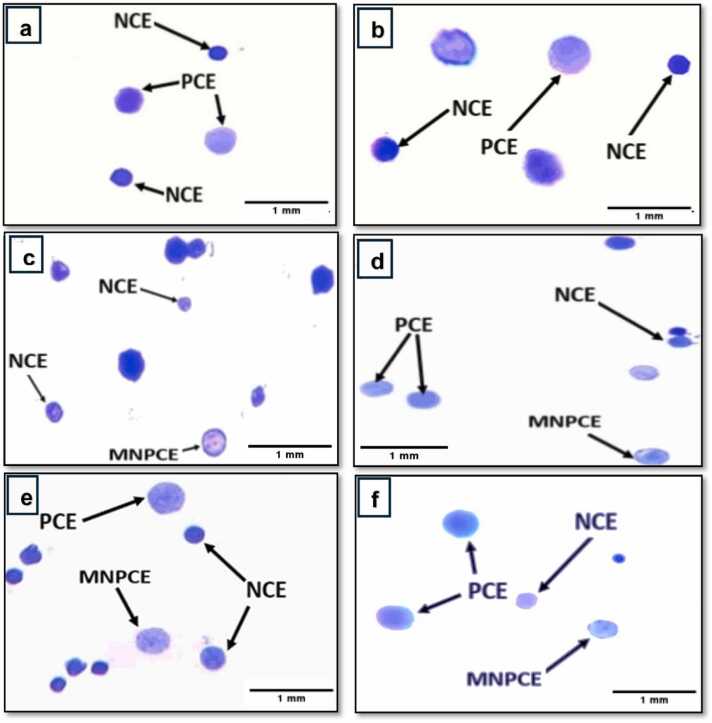
Bone marrow smears of male albino mice (*Mus musculus*) illustrating polychromatic erythrocytes (PCE), normochromatic erythrocytes (NCE), and micronucleated polychromatic erythrocytes (MNPCE). (a) Control group, (b) Vitamin D treated group, (c and d) low dose CAP treated group, (e and f) Vitamin D + low dose CAP treated group. The scale bar is 1 mm.

The results of the micronucleus test are depicted in Table 1. As shown in Fig. 3, the normochromatic erythrocytes (NCEs) appeared with a dark blue stain, while the polychromatic erythrocytes (PCEs) exhibited a light blue to violet stain. Micronucleated polychromatic erythrocytes (MNPCEs) were identified as polychromatic erythrocytes containing one or more small nuclei with a dark blue ring-shaped appearance, representing acentric fragments of chromosomes that lagged during the anaphase stage of cell division.

**Table 1:** Micronucleus assay results in bone marrow cells of male albino mice (n=3/group), treated with chloramphenicol (CAP) low dose and high dose (250 and 500 mg/kg b. wt.) and vitamin D (800 IU/kg) for 4 weeks (5 days/week). 6000 polychromatic erythrocytes (PCEs) and corresponding normochromatic erythrocytes (NCEs) were scored. Table shows mean ± standard deviation of micronucleated polychromatic erythrocytes (MNPCEs) and PCEs/NCEs ratio for control group and all treated groups.

Group No.	Treatment (Dose) mg/kg	Total observed cells/ No. of mice	Total MNPCEs	% Micronuclei (MNPCEs/ Total PCEs) (Mean± SD)	Total NCEs	Cytotoxicity PCEs/ NCEs (Mean± SD)
1	Control	6000/3	120	2 ±0.05000	5660	1.0609 ±0.01661
2	800 IU/kg vit D	6000/3	135	2.12 ±0.101	5750	1.0421 ±0.0735
3	250 mg/Kg b. wt. CAP	6000/3	194	3.2333 ±0.07638**	6560	0.913 ±0.0757
4	800 IU/kg vit D + 250 mg/kg b. wt. CAP	6000/3	154	2.5667 ±0.15899*	5990	1.0067 ±0.11060
5	500 mg/Kg b. wt. CAP	6000/3	394	6.57 ±0.51072**	7210	0.8333 ±0.03512**
6	800 IU/kg vit D + 500 mg/kg b. wt. CAP	6000/3	295	4.9167 ±0.38837**	6770	_0.8867 ±_0.06028**

* The results were as follows: Significant (P < 0.05) and * Significant (P < 0.01).

Mice subjected to 800 IU/kg vitamin D followed by a low dose of CAP orally exhibited a significant (P < 0.05) increase in the frequency of micronucleated polychromatic erythrocytes (MNPCEs) compared to the control group. This provides further evidence of the association between this treatment combination, as the group treated solely with vitamin D did not display an increase or presence of micronuclei. In contrast, the group treated with a low dose in the absence of vitamin D showed a significant (P < 0.01) augmentation in the occurrence of MNPCEs when compared to the control group. Furthermore, the administration of 800 IU/kg vitamin D prior to the administration of a high dose of CAP showed a significant (P < 0.01) elevation in the occurrence of micronucleated polychromatic erythrocytes. Treatment of mice with the high dose of CAP alone also showed a significant (P < 0.01) increase compared to the control group, with a notable difference in (Mean ± SD) being highly observable between the two groups.

**Fig. 4:**
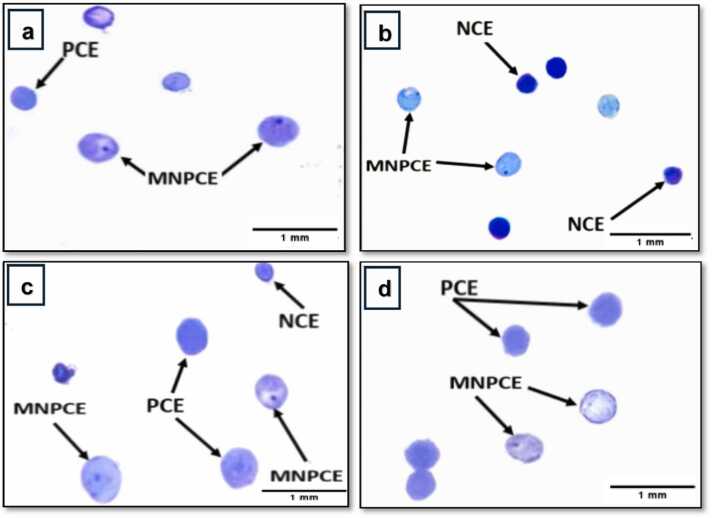
Photomicrograph of bone marrow smear of male albino mice *Mus musculus* showing polychromatic erythrocytes (PCEs), normochromatic erythrocytes (NCEs), and micronucleated polychromatic erythrocyte (MNPCE). (a and b) High dose (500 mg/kg b. wt.) treated groups, (c and d) Vitamin D + high dose (500 mg/kg b. wt). The scale bar is 1 mm.

Moreover, the protective function of vitamin D against CAP-induced cytotoxicity was evaluated through the PCEs/NCEs ratio, highlighting the hindrance or facilitation of the erythropoiesis process. Mice treated with 800 IU/kg vitamin D before the low dose of CAP showed a significant (P < 0.05) reduction in the mean compared to the control group. Conversely, the administration of both high doses of CAP, with and without vitamin supplementation, resulted in differences in the (Mean± SD) values of (0.8867±0.06028) and (0.8333±0.03512) respectively. This disparity demonstrated a significant (p<0.01) impact on cytotoxicity, as evidenced by a more pronounced decrease in the PCE/NCE ratio compared to the control group (Fig. 4).

## 4 Discussion

In the present study, we examined the safeguarding impact of vitamin D against the genotoxic and cytotoxic effects of chloramphenicol. This was assessed by measuring DNA fragmentation in liver tissue and the frequency of micronuclei formation in the bone marrow cells of male albino mice (*Mus musculus*)*.* Vitamin D was chosen due to its greater abundance in human nutrition, and lifestyle, and its favorable outcomes as demonstrated in the recent study of Sanlier et al. [30], vitamin D demonstrates efficacy in regulating hormone secretion, immune functions, and cell proliferation, as well as differentiation. It’s role as an immune modulator stems from the presence of receptors on various immune cells and the synthesis of its active metabolite within these cells. Acting as an immune system modulator, vitamin D suppresses cell proliferation and promotes cell differentiation [31].

Vitamin D may also provide protection against reactive oxygen species, which have the potential to induce DNA damage and chromosomal instabilities.

The results of the current investigation indicate that vitamin D demonstrated a cytoprotective role against the toxicity of CAP in bone marrow cells, evidenced by a significant (p < 0.01) reduction in the mean DNA fragmentation compared to mice administered with CAP alone. In a 2009 master's thesis, the impact of various forms of vitamin D, including cholecalciferol, was investigated on two osteosarcoma cell lines (SaOS-2 and 143B). Chloramphenicol (CAP) functions as a cytotoxic agent, inducing exogenous DNA damage through its chemical properties. CAP interacts with cellular components, especially under high concentrations, leading to DNA fragmentation, helix destabilization, and strand breaks. The compound can stimulate reactive oxygen species (ROS) formation, which causes oxidative stress and further DNA damage, amplifying the risk of genotoxicity [32]**.** This cytotoxicity is particularly notable as CAP can break down double-stranded DNA, a response seen in some in vitro studies where degradation intensified in the presence of reducing agents and metal ions. The study aimed to assess the dose-response relationship of vitamin D on cell differentiation, proliferation, and apoptosis. The findings indicated that higher concentrations of the vitamin significantly reduced cell proliferation at p≤0.05 in SaOS-2, while no significant effect was observed on 143B at 96 hours after vitamin administration [33].

The administration of vitamin D has demonstrated the ability to decrease oxidative stress-induced damage, prevent chromosomal aberrations, hinder telomere shortening, and inhibit telomerase activity in bot h animal models and cell lines [34]. This was also supported in a recent study that states that vitamin D changes expression of DNA repair genes in the patients with multiple sclerosis [35]. Hossein-nezhad et al. [36] also confirmed the impact of vitamin D levels and vitamin D3 supplementation on gene expression in healthy adults. Nineteen of these genes, influenced by vitamin D, have previously been shown to be regulated by 1,25-dihydroxyvitamin D3 [1,25(OH)₂D₃] in vitro, and they play roles in the immune system, apoptosis, transcription regulation, and stress response.

The micronucleus test has been incorporated into numerous studies to identify the genotoxic effects of various classes of chemicals in mammalian systems [37], In light of this, the present study utilized this test to assess genetic damage in bone marrow cells of treated male albino mice, comparing the results with those of the control group. Moreover, it has been acknowledged as the most dependable and extensively employed bioassay for evaluating DNA damage in mammalian cells *in vivo*. This is attributed to its capability to identify genomic alterations arising from chromosomal damage and/or impairment to the mitotic apparatus induced by clastogenic agents [38].

Micronuclei (MN) are minute chromatin bodies that emerge in the cytoplasm through the condensation of acentric chromosome fragments or entire chromosomes. Typically, they are provoked by clastogenic substances or spindle poisons during the division of cells, particularly in tissues like bone marrow. The frequency of micronuclei has been widely acknowledged as a dependable indicator for identifying chromosome breakages and losses [39]**.**

The anti-inflammatory, anti-oxidant property of vitamin D is evident in groups treated with vitamin D+ CAP in both cases studied where the group treated with vitamin then a low dose of the antibiotic showed a decrease in the mean value by 0.6333 and administration of vitamin D with CAP (500 mg/kg) showed a decrease of 1.6533 in the mean value. It has been validated that vitamin D possesses robust anti-inflammatory properties, leading to a decrease in pro-inflammatory mediators and an elevation in anti-inflammatory cytokines. Additionally, there is evidence suggesting that vitamin D may reduce C-reactive protein (CRP) levels and impact certain hematological indices [40], [41], [42]**.** Also, Hayder [43] in his recent study stated that vitamin D3 has been shown to decrease the frequency of micronucleus formation in normal bone marrow stem cells and reduce the serum levels of the P53 protein. Çelik [44] reported the inhibition of immature erythrocytes (PCE) in comparison to mature erythrocytes (NCE) leads to a reduction in the PCE to NCE ratio. This ratio serves as a crucial indicator of cytotoxicity and is routinely incorporated into micronucleus tests for evaluating the mutagenic effects of chemicals on mammals. In this study, the oral administration of vitamin D prior to chloramphenicol (CAP) resulted in a reduced frequency of micronuclei and an increased ratio of polychromatic erythrocytes (PCEs) in the bone marrow of treated rats compared to the control group. When administered without vitamin D, CAP induced cytotoxicity, evidenced by an elevated PCE to NCE ratio. The findings indicate that the mean PCE/NCE ratio was significantly reduced (p < 0.01) by the combined administration of CAP and vitamin D, suggesting the protective role of vitamin D against CAP-induced cytotoxic effects on bone marrow tissue.

The myelotoxicity observed was particularly pronounced in the erythroid series. Nevertheless, the toxic effects were not confined to the erythroid lineage, as evidenced by some reductions in colony-forming unit granulocyte-macrophage (CFU-GM) counts and peripheral blood leukocyte counts at different cigarette smoke condensate (CAP) doses. Additionally, while cytoplasmic vacuolation was evident in cells of the erythroid series, precursors of the myeloid lineage were also affected [45]**,** It is assumed that vitamin D plays a pivotal role as a regulator of oxidative stress and inflammatory parameters [46] reinforcing the results obtained in the current study, as it led to a significant (P < 0.01) decrease in the ratio of PCE to NCE.

The latest findings from the micronucleus test revealed a significant increase (p < 0.05) in the mean of micronucleated polychromatic erythrocytes (MNPCEs) in mice following the oral administration of CAP (250 mg/kg) for five consecutive days a week over a period of 4 weeks, as compared to the corresponding control group as mentioned by Yunis and Adamson [47], chloramphenicol induced a dose-dependent inhibition of erythroid and granulocytic colony-forming units derived from LA-1 mice. This was supported by Shukla et al. [48] as they demonstrated that substances like p-nitrosulfathiazole, known as chloramphenicol derivatives, can inhibit DNA synthesis in marrow stem cells, ultimately leading to cell death. Chloramphenicol is linked to sideroblastic bone marrow changes. It primarily inhibits erythropoiesis in a consistent and dose-dependent manner. This effect is distinct from and unrelated to the rare complication of aplastic anemia. Sideroblastic anemias are characterized by the presence of a variable number of hypochromic cells in the peripheral blood, along with an excess of iron in the bone marrow. This leads to the development of erythroblasts containing iron granules arranged in a ring around the nucleus, resulting in ineffective erythropoiesis [49]. Furthermore, the micronucleus test revealed a significant increase (p < 0.01) in the mean of micronucleated polychromatic erythrocytes (MNPCEs) in mice following the oral administration of CAP (500 mg/kg) with and without vitamin supplementation for four consecutive days, as compared to the corresponding control group indicating CAP is known to be a contributing factor in the aplastic anemia for animals, e.g., rat, cat, dog, and pig and humans [50].

While vitamin D was initially recognized for its crucial role in calcium homeostasis and its necessity for proper bone formation, current research focuses extensively on studying vitamin D signaling within the immune system [51]**,** Carlberg [52] found that the majority of genes targeted by vitamin D are typically down-regulated, especially when cells are stimulated with 1,25(OH)2D3 for 24 hours or longer, vitamin D tends to counteract the up-regulation of genes rather than significantly down-regulating their expression [53]**,** vitamin D receptor (VDR) functions by opposing pro-inflammatory transcription factors like NFAT, AP1, and NF-κB in immune cells [54]. This mechanism could be the reason why vitamin D reduces the toxic effects induced by chloramphenicol (CAP). Vitamin D's ability to counteract pro-inflammatory factors may contribute to its protective effect against the toxic impact of CAP.

## 5 Conclusion

Results of the present investigation revealed that vitamin D, acting as a geno- and cyto-protective agent abundant in food sources and sunlight, effectively protects against mutagens like chloramphenicol (CAP). Vitamin D significantly reduced DNA damage in liver cells and cytotoxic effects in bone marrow cells of male albino mice treated with CAP. Hence, individuals undergoing treatment with CAP and its derivatives are advised to incorporate vitamin D into their diet or consider supplements. This is recommended to counteract the prolonged impact on bone marrow associated with the use of CAP and its derivatives.

## CRediT authorship contribution statement

**Asmaa Ahmed Khaled Emam:** Writing – review & editing, Supervision. **Mahmoud Fathy Mahmoud:** Supervision. **Omnia Nabeel Mohamed Morgan:** Writing – original draft, Visualization, Methodology. **Sally Ramadan Gabr Eid El-Ashry:** Writing – review & editing, Validation, Supervision, Methodology, Investigation, Conceptualization. **Nagla Zaky Ibrahim El-Alfy:** Writing – review & editing, Validation, Supervision, Investigation, Conceptualization.

## Declaration of Competing Interest

The authors declare that they have no conflict of interest in the research presented in this paper.

## Data Availability

Data will be made available on request.
